# Robotic-assisted minimally invasive esophagectomy: experience at the University of Pittsburgh Medical Center

**DOI:** 10.20517/2574-1225.2025.51

**Published:** 2025-06-24

**Authors:** Marissa A. Matto, Evan T. Alicuben, Samuel Luketich, Sangmin Kim, Nicholas Baker, Inderpal S. Sarkaria, James D. Luketich

**Affiliations:** 1 Department of Cardiothoracic Surgery, University of Pittsburgh Medical Center, Pittsburgh, PA 15232, USA.; 2 Department of Cardiovascular and Thoracic Surgery, West Virginia University Medicine, Morgantown, WV 26506, USA.; 3 Department of Cardiovascular and Thoracic Surgery, UT Southwestern Medical Center, Dallas, TX 75390, USA.

**Keywords:** RAMIE, robotic esophagectomy, minimally invasive esophagectomy

## Abstract

Robotic-assisted minimally invasive esophagectomy (RAMIE) is increasingly used in the treatment of resectable esophageal cancer. This is a report on the current technique of RAMIE at University of Pittsburgh Medical Center (UPMC), including a summary of early data on 65 patient outcomes reported in an ongoing esophageal cancer database. To date, we have performed over 200 cases of RAMIE at UPMC from September 2013 to July 2024, and the analysis of the data will be presented soon. The practice has evolved into a near-total RAMIE experience for several surgeons, while others remain in a learning curve. It is our experience that the initial performance of RAMIE requires strong mentoring by an experienced robotic surgeon. However, at this time, we are unable to provide guidelines for specific case numbers to achieve proficiency. As more patients with esophageal cancer are treated with robotic-assisted minimally invasive esophagectomy (MIE) at UPMC, data have shown that patient outcomes are not compromised compared with that of traditional MIE. In fact, RAMIE may demonstrate superiority in the median number of lymph nodes harvested, which could contribute to increased accuracy in pathologic staging. This approach has developed a strong surgeon preference for both new graduates and experienced MIE surgeons alike.

## INTRODUCTION

Several recent reviews have shown that minimally invasive esophagectomy (MIE) has exceeded open esophagectomy in the United States. It has become the standard of care for resectable esophageal cancer in most tertiary care centers^[1]^. Work at the University of Pittsburgh has significantly contributed to this transition, and this institution’s experience has now exceeded 3,000 MIEs^[2–4]^. Robotic-assisted minimally invasive esophagectomy (RAMIE) has become increasingly used in the treatment of esophageal cancer in our center and across the world in recent years. Proponents of RAMIE cite many potential advantages, including a greater lymph node harvest compared to MIE, less postoperative pain, and a lower rate of pulmonary complications^[5,6]^. RAMIE has been shown to be equivalent in terms of anastomotic leak and 30-day mortality^[7]^, but there is minimal level 1 evidence available at this time.

Robotic technology provides 3D visualization, 8 degrees of rotation, and stabilization of tremor, all advantages in the small confines of the mediastinum. These advantages are reported anecdotally by many surgeons; however, in studies comparing laparoscopic and robotic surgeries, advantages in patient outcomes remain to be seen. The objective of this manuscript is to provide a detailed report on the current technique of RAMIE at the University of Pittsburgh Medical Center (UPMC), highlighting a novel anastomotic technique and a brief review of our previously published data.

## OPERATIVE TECHNIQUE

### Abdominal approach

The most common robotic approach performed at UPMC is the Ivor Lewis esophagectomy, which has been previously described^[5]^. Patients are initially placed supine with a footboard in place. Prior to incision, upper endoscopy and bronchoscopy are performed. Four robotic ports and two non-robotic ports are used. One non-robotic port is an assist port (12 mm) in the lower right quadrant, next to the umbilicus. The other non-robotic port is a 5 mm port along the lateral lower right costal margin for the Mediflex liver retractor. From the patient’s position, the robotic port configuration is as follows: From right to left, arm 1 has the force bipolar grasper (which can be exchanged for the stapler), arm 2 holds the 30-degree camera, arm 3 has either an ultrasonic shear or the spatula, and arm 4 has an atraumatic bowel grasper. The lower right quadrant non-robotic port is a standard laparoscopic assistant port, through which the assistant primarily uses a suction or grasper. The right robotic port is 12 mm, and the remaining ports are 8 mm. These ports are in similar locations reported previously^[5]^.

**Figure 1.**
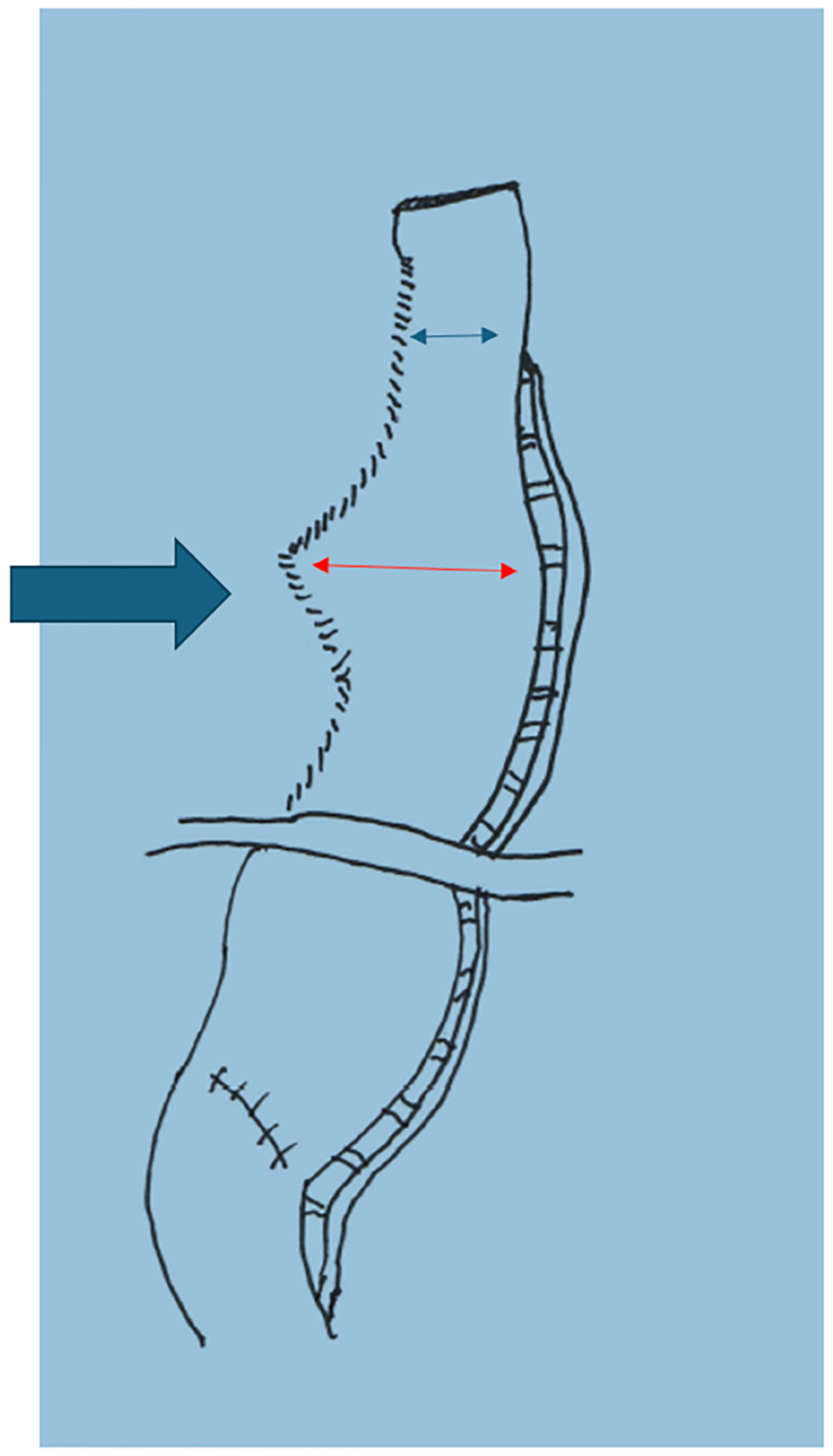
Newly created gastric conduit with widened area shown (red arrow) for insertion of EEA handle. The diameter of the red arrow measures approximately 4.5 cm and the diameter of the blue arrow (our preferred conduit width) is 3 cm. EEA: End-to-end anastomosis.

**Figure 2.**
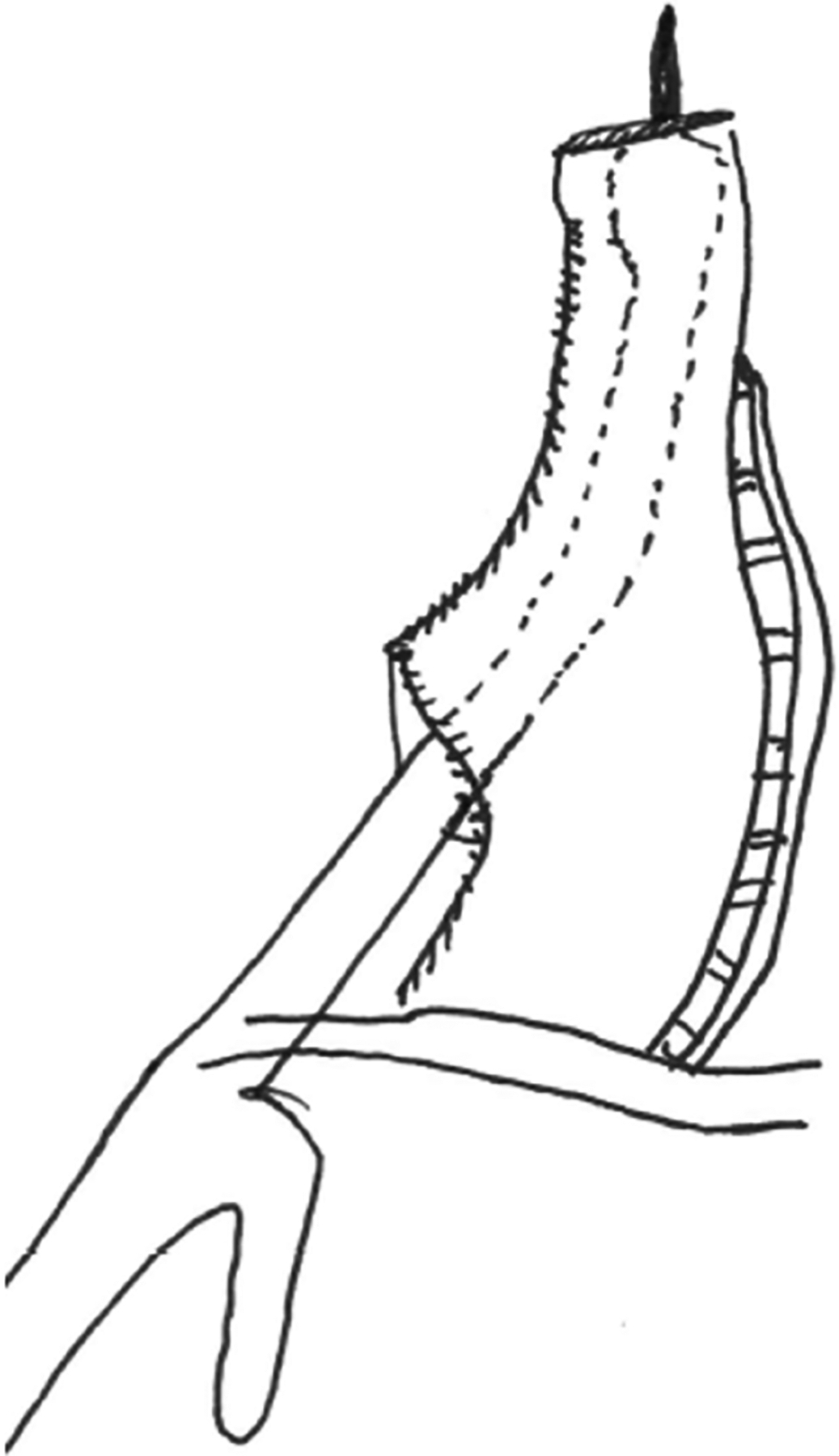
EEA handle inserted through the widened conduit area to facilitate a true EEA with the proximal esophageal anvil. EEA: End-to-end anastomosis.

**Figure 3.**
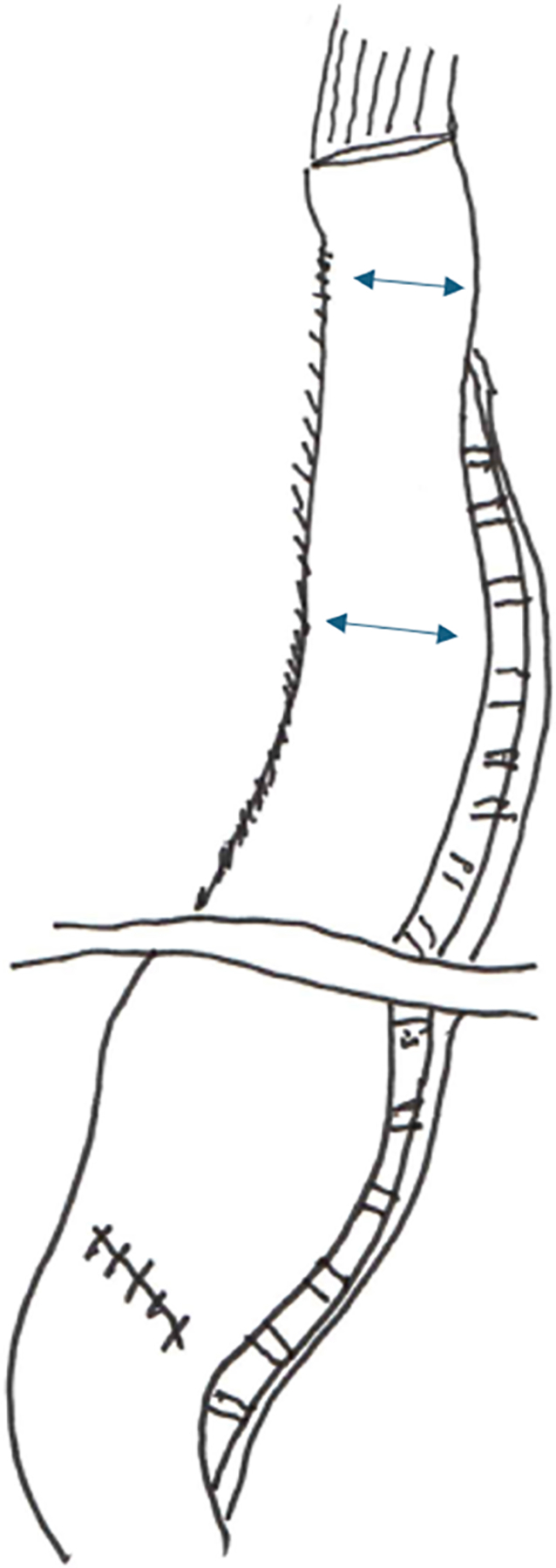
Completed true EEA (intrathoracic esophagogastric) with the restapled area forming a uniform 3-cm-wide conduit. Arrows indicate the location of the 3-cm-wide conduit. EEA: End-to-end anastomosis.

The procedure begins with exposing the crus by dividing the gastrohepatic ligament. The esophagus is then mobilized by incising the phrenoesophageal ligament. The dissection then proceeds to expose the left gastric artery pedicle by entering the lesser sac. A complete celiac lymphadenectomy is then performed. The short gastric and left gastric arteries are then divided, taking care to preserve the right gastroepiploic artery. At this point, indocyanine green (ICG) and near-infrared fluorescence can be used as needed to identify the gastroepiploic arcade. This technique is not yet our standard of care but is part of an ongoing investigation. An omental flap can also be created at this point if desired. Our current practice is to use a formal omental pedicled flap only for cases of preop neoadjuvant chemotherapy and radiation. In other cases, an approximately 3 cm margin of omental fat is left protecting the gastroepiploic and placed between the conduit and airway. Recently, we have added a “widening” to the gastric conduit [Figure 1] to enable the introduction of the end-to-end anastomosis (EEA) stapler handle through the lesser curvature staple line [Figure 2]. After the instrument is docked and the EEA anastomosis is completed in the chest, the EEA device is removed, and the widened part of the conduit is restapled to conform to the 3 cm final width of the conduit [Figure 3]. This creates a true EEA. After ensuring complete gastric mobilization from the hiatus to the pylorus, the conduit can be created with multiple firings of the stapler [Figure 1]. This should be done parallel to the greater curvature of the stomach. We insert a ruler into the abdomen to measure the width of the conduit to be approximately 3 cm. We then secure the specimen to the conduit for ease of transport into the thoracic cavity. If an omental flap was created, we also secure it to the conduit. We then perform a Heinecke-Mikulicz pyloroplasty. We have recently completed a randomized trial on pyloroplasty during MIE and will present our results in an upcoming surgical meeting. While many other surgeons perform no pyloric intervention, balloon dilation of the pylorus, or Botox injection, our standard practice is to perform a pyloroplasty. To conclude the abdominal portion, we place a feeding jejunostomy tube laparoscopically, using our standard laparoscopic J-tube approach^[7]^. While some surgeons are performing the jejunostomy with the robot, we prefer a laparoscopic approach. Prior to the completion of the abdominal phase of the RAMIE procedure, we place marking stitches to indicate the desired position of the lower border of the newly created conduit relative to the diaphragmatic hiatus, in order to avoid pulling up too much conduit. The marking stitch has been sufficient for accurate positioning in most circumstances.

### Thoracic approach

**Figure 4.**
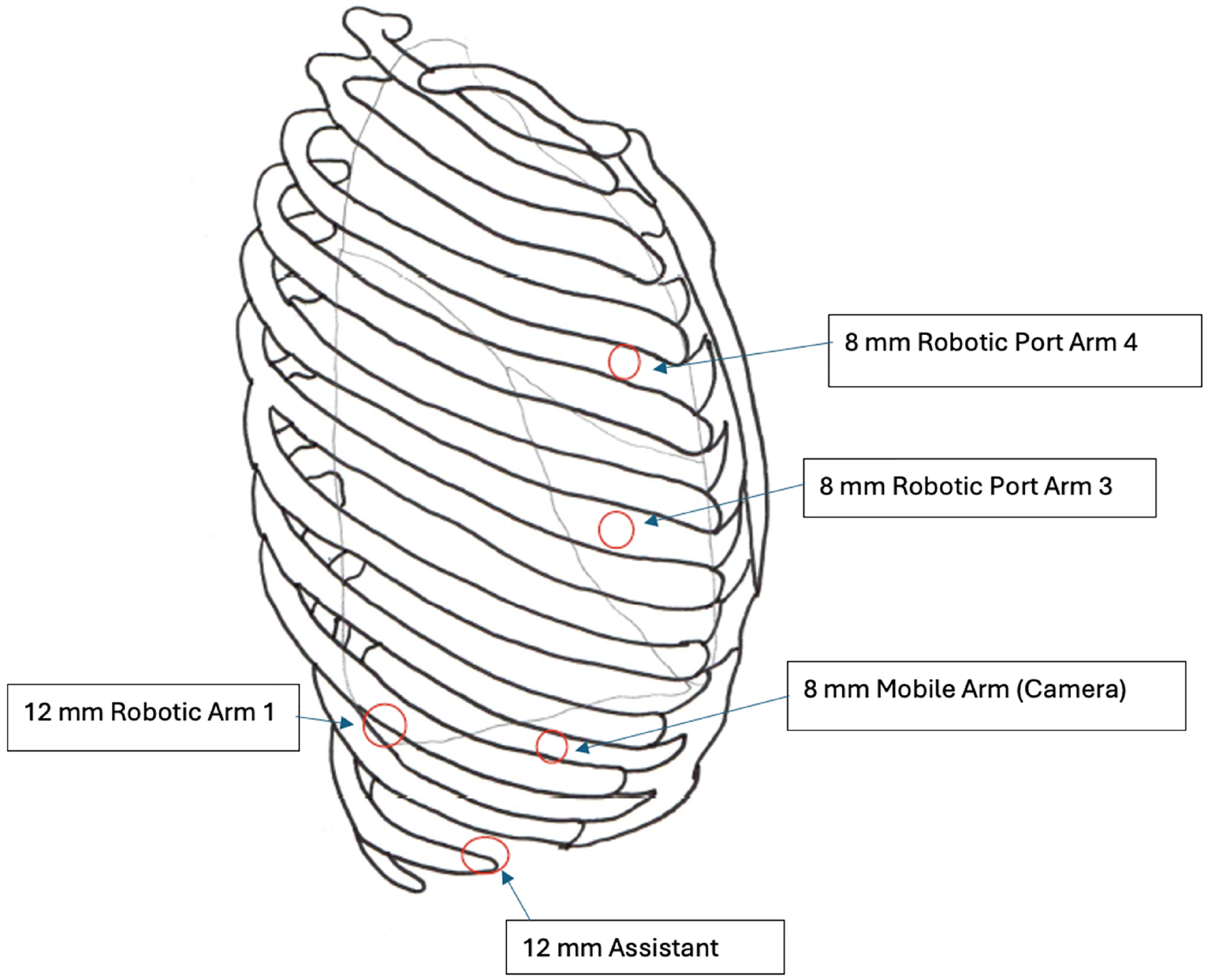
Port placement for thoracic portion of RAMIE. RAMIE: Robotic-assisted minimally invasive esophagectomy.

For the thoracic portion, we use 4 robotic ports and 1 non-robotic port [Figure 4] for the chest. After positioning the patient in the left lateral decubitus position, we access the thoracic cavity through the third or fourth intercostal space. We chose this site to avoid the diaphragm, and it will ultimately serve as a robotic port. Once the thoracic operative field is established, any adhesions present are divided using a video-assisted thoracoscopic surgery (VATS) approach, enabling safe placement of the remaining ports. The 8 mm robotic camera port is placed over the ninth rib in the eighth intercostal space, along the mid-axillary line, under direct vision. The next 8 mm robotic port is placed in the fifth or sixth intercostal space (at least two interspaces above the camera port) along the same line, which is near the mid- to anterior axillary line. The final robotic port, a 12 mm port, is placed in the ninth to tenth intercostal space, posterior to the camera port and at least 8 cm away from it. The final location of all robotic ports may be adjusted based on the patient’s body habitus. In addition, it is important to maintain close to 8 cm between each robotic port. From the patient’s right to left, robotic arm 1 has the force bipolar (later exchanged for the robotic stapler), robotic arm 2 holds the 30-degree robotic camera, arm 3 has either the ultrasonic shear or an energy dissector, and arm 4 has an atraumatic grasper. The assistant port is placed between the inferior working ports and as low as the costal margin-diaphragm interface will allow. This 12 mm port serves as a second 12 mm robotic port but is mostly used as a standard VATS port for suctioning, suture and sponge insertion, and small specimen retrievals. We use it for stapling if the angle appears superior to robotic arm 1.

During the thoracic phase, a crucial principle is to achieve complete circumferential mobilization of the esophagus. This mobilization extends from the hiatus up to the level of the azygous vein. Throughout this process, meticulous attention is given to harvesting all periesophageal lymph nodes. This comprehensive approach ensures thorough dissection and optimal lymph node retrieval, which are essential for both staging and potential therapeutic benefits in esophageal cancer surgery. For maximum safety, we dissect from a known area, e.g., the pericardial interface with the esophageal mediastinal pleural border, into areas less clearly identified. A good initial dissection plane is along the lower esophageal margin, opening the mediastinal pleura and mobilizing the inferior pulmonary ligament. Avoiding even minute injuries to the inferior pulmonary vein is important. The phrenic nerve should be readily visible and is generally easy to avoid. As this dissection progresses, we typically start with the spatula. When encountering significant aortoesophageal branches and/or bronchial arteries, we alternate between the spatula and robotic ultrasonic shears to achieve optimal hemostasis. Special care is taken when dissecting the subcarinal lymph nodes, minimizing energy use to reduce the risk of airway injury. Attention is also given to preserving the thoracic duct, aorta, and azygous vein branches. We often extend the dissection plane superiorly along the carina, bronchus intermedius, right upper lobe bronchus, and beyond the azygous vein. Once above the azygous vein, the dissection is kept as close to the esophagus as possible to avoid unnecessary injury, as most GE junction tumors do not require extensive lymph node dissections beyond this level for an R0 resection. If preoperative imaging suggests disease in this area, additional caution is exercised. As the dissection ascends, particular attention is required to avoid injury to the airway and the recurrent laryngeal nerves. During the posterior mediastinal dissection along the azygous vein, we employ a gentle technique to separate the esophagus and associated lymph nodes from the aorta and thoracic duct, taking great care to avoid injury to these vital structures. Our approach alternates between anterior and posterior planes as necessary, ensuring the esophagus and lymph nodes remain *in situ* while working from side to side. At some point, we connect this mediastinal pleural plane inferiorly near the diaphragm, extending laterally toward the chest wall, descending aorta, and azygous vein, forming a “U-shaped Pathway”. This exposes the distal esophagus and proximal stomach and facilitates specimen retrieval into the chest.

Once the specimen is visible in the chest, the tip of the gastric conduit can be seen sutured to the staple line along the lesser curvature of the stomach. We routinely suture the top of the new gastric conduit, along the greater curvature vessels, to the specimen’s staple line near the lesser curvature vessels and associated fat. At this point, it is essential to carefully assess the orientation of the gastric conduit as it is brought into the chest. This helps prevent spiraling and ensures proper orientation. The surgeon should confirm that the staple line of the neo-esophagus (gastric conduit) is facing the right thoracic cavity, while the greater curvature and short gastric vessels are directed downward, toward the spleen. Once proper orientation is confirmed, the suture connecting the esophagogastric specimen to the new gastric conduit is divided. A single holding stitch is then placed at the tip of the gastric conduit to secure it to the diaphragm, thereby maintaining correct orientation and preventing retraction into the abdominal cavity.

The esophageal dissection should extend proximally to a point where the surgeon is confident that an adequate proximal margin will be achieved. It is important to consider the original tumor location prior to any neoadjuvant therapy. We generally aim for at least a 5 cm gross margin from the proximal edge of the tumor and/or Barrett’s esophagus to the point of esophageal transection. It is generally good practice to have a flexible esophagoscope in place, allowing an assistant to confirm that the planned transection site will provide the desired margin. Before finalizing the transection site, the surgeon should have already performed an on-the-table esophagogastroduodenoscopy to view the esophagus and tumor at the beginning of the case. Based on these findings, the surgical team should have a discussion to reach a consensus on the optimal transection point. Before dividing the proximal esophagus, the gastric conduit should be retrieved and the tip carefully lifted to the anticipated site of the proximal esophageal anastomosis. In most GE junction tumors, there is usually sufficient length in the tension-free conduit to permit resection of several centimeters of the gastric conduit tip while still reaching the proximal esophagus margin above the azygous vein. If there is concern about conduit tension or difficulty reaching the planned transection site, several considerations must be addressed before proceeding with esophageal division or stapling the conduit tip.

In cases of conduit tension, the surgeon should first carefully examine the orientation of the conduit and gently draw the greater curvature fat and omentum into the thoracic cavity. The greater curvature fat, especially when an omental pedicle flap is present, can often become trapped at the hiatus. Careful dissection in this area can frequently release the conduit, facilitating its advancement into the chest. At this stage, it is also important to avoid drawing excessive gastric conduit into the thoracic cavity, as this can create a redundant segment just above the hiatus, potentially leading to dysphagia and stasis within the conduit.

If tension-free delivery of the conduit tip remains problematic, surgical judgment becomes critical in considering leaving more proximal esophagus. Performing an anastomosis under tension significantly increases the risk of leakage and stenosis. Another option is to retain a longer segment of the conduit by resecting less of its tip. Ideally, a good oncologic margin can still be achieved while removing 5 or more centimeters of the conduit tip. This resection eliminates potentially ischemic tissue and provides additional length at the gastric margin. A thoughtful and meticulous approach to these steps is crucial to achieving both optimal oncologic and functional outcomes.

After dividing the proximal esophagus, the specimen is sent for frozen section analysis to assess the surgical margins. The conduit and proximal esophageal end are then prepared for anastomosis. If there is any concern regarding the margins, we halt the procedure and wait for the frozen section results. If the margins are confirmed to be negative, we proceed with the initial steps of the anastomosis. We begin by evaluating the internal diameter and compliance of the proximal esophageal end. In most cases, this is located well above the level of the azygous vein division, and the open esophageal lumen is wide enough to accommodate a 28 mm anvil, which can be inserted and sutured without difficulty. If the proximal esophagus is narrow, we may consider using a 25 mm anvil or performing a handsewn anastomosis. We avoid using a 21 mm anvil in adults, as this has consistently led to refractory strictures and persistent dysphagia. In many patients, distal obstruction caused by the tumor results in prestenotic dilation of the proximal esophagus, facilitating the insertion of a 28 mm anvil. However, long-standing GERD or prior radiation therapy can lead to a very stiff and narrowed mid-to-proximal esophagus. To assess the internal diameter, the esophageal lumen is gently spread using an endoscopic sponge stick or a similar instrument. Occasionally, a 30 cc Foley balloon may be inserted into the lumen for gentle dilation. If all attempts to insert an anvil of the intended size fail, we either reduce the anvil size, opt for a handsewn anastomosis, or consider a linear stapled technique. Once the anvil is in place, a purse-string suture with 2–0 Ethibond is used to secure the esophageal edges around the anvil post. A second purse string is then placed to further secure the tissue and create a flat esophageal surface for stapling. This step helps ensure the formation of complete “rings” during the EEA stapling.

Once securing the anvil, we proceed with the insertion of the EEA stapler through the robotic arm 1 port site. Since the EEA stapler is not compatible with the robotic system, the port must be temporarily removed and the incision enlarged to accommodate the device. Previously, our technique involved inserting the EEA shaft through the end of the open conduit, exiting through the posterior wall and along the greater curvature, before docking with the anvil. The stapler would then be fired, and the remaining open end of the conduit stapled closed. Over time, this approach has evolved into a stapled EEA, as described earlier [Figures 1–3].

Most Ivor Lewis anastomoses are performed above the level of the azygous vein. Even in cases involving small GE junction tumors, we rarely consider a low-lying gastroesophageal anastomosis, as this invariably leads to severe and potentially recalcitrant bile and acid reflux. Such reflux carries an inherent risk of developing Barrett’s esophagus and second malignancies. Therefore, to achieve optimal surgical margins and functional outcomes that minimize reflux symptoms, we prefer a narrow gastric conduit with the anastomosis placed above the azygous division point, avoiding any redundancy or spiraling.

After the anastomosis, a perianastomotic drain and a nasogastric tube (NGT) are placed. At this stage, the chest is irrigated with several liters of warm antibiotic solution to remove any spillage of esophageal or gastric contents. We then inject approximately 20 mL of 0.5% bupivacaine with epinephrine into the intercostal spaces from the third down to the eleventh, administering approximately 2 cc per interspace. Next, we carefully position the greater curvature fat and epiploic arcades between the conduit and the mediastinal airways and pericardium. We prefer to orient the staple line away from the airway, with the greater curvature fat and associated vessels facing the deeper mediastinum. The tip of the drain is then loosely secured with a 4–0 absorbable suture to a delicate 1 mm edge of the mediastinal pleura. This suture helps prevent malposition of the drain tip during lung expansion and postoperative mobilization. A flat Jackson-Pratt drain is used and connected to either a bulb or bile bag for drainage.

Finally, an endoscope is passed to check for anastomotic leaks and to ensure the gastric conduit lies without shelf formation or redundancy. If a shelf is detected, we attempt to carefully push the conduit further into the abdominal cavity. Should these efforts fail and a significant abnormal shelf is identified near the thoracoabdominal junction, we consider performing laparoscopy at the conclusion of the thoracic procedure. The patient is repositioned supine, and a laparoscope is introduced. This can be done laparoscopically, though the robotic system may be redocked if needed. If re-entry into the abdomen is required, we further reduce and straighten the redundant gastric conduit and secure it in place from below. This step is rarely necessary when careful attention is paid to conduit measurement and delivery during the initial anastomosis construction.

### Postoperative protocols

While this is primarily a technique paper, we would like to share some unpublished details regarding our general postoperative management of RAMIE patients. Ideally, patients are extubated in the operating room immediately following surgery. If the anesthesia team determines that the patient is not ready, extubation is delayed until postoperative day (POD) 1. A NGT is placed at the end of the procedure and typically removed on POD1. After NGT removal, patients may have minimal ice chips (approximately 1 teaspoon) every 2–3 h, assuming they pass a bedside swallow evaluation. On POD3, a barium swallow study is conducted. If concerns arise during the bedside evaluation, a modified barium swallow may be performed to test for aspiration. The patient remains NPO until they demonstrate safe swallowing without coughing or signs of aspiration. If the patient passes the barium swallow without evidence of a leak, they may begin with 1–2 oz of clear liquids and advance rapidly over the following days to a soft diet (approximately 3–4 oz every 4 h). During this transitional period, the primary source of nutrition is via jejunostomy tube feeds.

Regarding ambulation, the patient is assisted out of bed to a chair on the morning of POD1. If overall progress is satisfactory, the patient is transferred to a monitored unit and begins assisted ambulation, potentially as early as POD1. Adjustments to ambulation are made based on the patient’s clinical status.

## RESULTS

In a previously published, propensity-matched comparison of MIE and RAMIE, we reported on 181 MIE patients and 65 RAMIE patients treated between 2014 and 2021^[5]^. The primary endpoints of this retrospective study were overall survival and disease-free survival. Kaplan-Meier curves demonstrated comparable outcomes between MIE and RAMIE, with *P*-values of 0.69 and 0.70, respectively.

A total of 83 patients underwent RAMIE during the study period, of whom 65 were included in the detailed propensity-matched analysis. These cases were relatively evenly distributed across the study period, with a slight increase in the last 3 years compared to the first 4 years. Initially, only one surgeon performed RAMIE; over time, three additional surgeons were credentialed and began performing the procedure. The inclusion and exclusion criteria for the cohort and matched subset were previously described^[5]^. In brief, inclusion criteria encompassed all patients who underwent MIE or RAMIE. Exclusion criteria included patients over 87 years of age, those with metastatic disease or cirrhosis, and patients who underwent bipolar exclusion or hybrid procedures. Table 1 summarizes patient demographics. The median age was 67 years, and the majority were male (81.5%). Most patients presented with clinical stage III disease (67.7%) and had adenocarcinoma (86.2%). Perioperative outcomes for RAMIE are presented in Table 2. The median number of lymph nodes removed was 32, significantly higher than the 29 removed in MIE cases (*P*-value = 0.02). An R0 resection was achieved in 64 patients (98.5%). The median operative time for RAMIE was 614 min, which did not significantly differ from that of MIE (625 min; *P*-value = 0.86). The 90-day mortality rate was 3.1% (2 patients) for RAMIE and 2.4% (4 patients) for MIE (*P*-value = 0.73). Major morbidities associated with RAMIE are listed in Table 3. Compared with MIE, there were no statistically significant differences in major morbidities, including pneumonia (*P* = 0.34), atrial arrhythmia requiring treatment (*P* = 0.19), and anastomotic leaks graded ≥ 3 (*P* = 0.49).

## DISCUSSION

This manuscript describes our current RAMIE technique at UPMC. The data suggest that RAMIE can be performed with favorable outcomes at experienced institutions^[5]^. These outcomes are comparable to those reported in previous studies of MIE, particularly in terms of mortality rates, adequacy of oncologic resection, and perioperative complications^[5]^. This suggests that both patient and oncologic outcomes are not compromised by the robotic approach.

RAMIE may be superior to MIE in the median number of lymph nodes harvested - 32 *vs*. 21^[4,5]^. This difference likely reflects one of the key benefits of robotic surgery - enhanced surgical control and ease of tissue dissection in anatomically challenging regions. Robotic technology provides surgeons with improved visualization and greater dexterity for complex maneuvers^[8]^. Harvesting a larger number of lymph nodes may improve long-term oncologic outcomes by enabling more accurate pathological staging. While these findings are encouraging, further studies are needed to determine whether the number of harvested lymph nodes correlates with recurrence rates.

Two recent studies from our institution involving 25 and 65 patients, respectively, demonstrated a reduction in median operative time from 661 to 614 min^[5,7]^. This suggests that procedural efficiency improves with increased surgeon and institutional experience. However, it remains unclear whether our results at a high-volume center can be replicated in lower-volume institutions treating fewer patients with resectable esophageal cancer. Given the complexity of this operation, having well-trained surgeons and staff is critical to achieving optimal outcomes. Therefore, the generalizability of our findings to less specialized centers may be limited. Future directions for our research include an analysis of over 200 RAMIE cases performed to date, as well as an evaluation of the cost-effectiveness of RAMIE compared to MIE.

## Figures and Tables

**Table 1.** Characteristics of patients undergoing RAMIE

Variable	*N* (%) or median (p25, p75)
*N*	65
Age	67 (60,72)
BMI	28.2 (24.6, 31.9)
Clinical stage	
I, IA, IB	4 (6.2)
II, IIA, IIB	13 (20.0)
III, IIIA, IIIB, IIIC	44 (67.7)
IV, IVA	4 (6.2)
Clinical T category	
T1, T1a, T1b	4 (6.2)
T2	9 (13.9)
T3	51 (78.5)
T4, T4a, T4b	1 (1.5)
Charlson comorbidity index	1.0 (0, 1)
GERD	
Yes	39 (60.0)
ASA score	
2	5 (7.7)
3	56 (86.2)
4	4 (6.2)
Clinical N category	
N0	19 (29.2)
N1	32 (49.2)
N2	11 (16.9)
N3	3 (4.6)
Neoadjuvant treatment	
CRT	39 (60.0)
CT	16 (24.6)
None	10 (15.4)
Drug use	
Never	62 (95.4)
Previously	1 (1.5)
Yes	2 (3.1)
Gender	
Male	53 (81.5)
Family history	
Yes	4 (6.2)
Smoking status	
Never	14 (21.5)
Current	43 (66.2)
Past	8 (12.3)
Tumor type	
Adenocarcinoma	56 (86.2)
Squamous cell carcinoma	9 (13.8)

RAMIE: Robotic-assisted minimally invasive esophagectomy; BMI: body mass index; GERD: gastroesophageal reflux disease; ASA: American Society of Anesthesiologists Physical Status Classification; CRT: chemoradiotherapy; CT: chemotherapy.

**Table 2.** Perioperative outcomes

Variable	*N* (%) or median (p25, p75)
Estimated blood loss (mL)	200
Total lymph nodes removed	32 (25, 39)
Completeness of resection	
R0	64 (98.5)
R1	1 (1.5)
Total operative time (min)	614 (562, 673)
30-day readmission	13 (20.6)
Length of stay (days)	8 (7, 13.5)
In-hospital mortality	2 (3.1)
30-day mortality	2 (3.1)
90-day mortality	2 (3.1)

**Table 3.** Major morbidity

Variable	*N* (%)
Pneumonia	9 (17)
Atrial arrhythmia requiring treatment	19 (38.8)
Anastomotic leak (grade ≥ 3)	3 (4.6)
Chylothorax requiring treatment	4 (8.3)
Recurrent laryngeal nerve paralysis	0 (0)
Intraoperative complications	6 (9.2)

## Data Availability

Data for this manuscript were taken from Ref.^[5]^.
